# Améloblastome du sinus maxillaire traité par radiothérapie

**DOI:** 10.11604/pamj.2017.26.169.8992

**Published:** 2017-03-24

**Authors:** Asmaa Chehal, Rosabel Lobo, Asmaa Naim, Ignacio Azinovic

**Affiliations:** 1 Centre Régional d’oncologie Al Hoceima, Maroc; 2 Instituto Madrileño de Oncologia, IM Oncology, Espagne; 3 Université Mohammed VI des Sciences de la Santé, Hôpital Cheikh Khalifa

**Keywords:** Améloblastome, maxillaire, tumeur, Ameloblastoma, maxillary, tumor

## Abstract

L’améloblastome est une tumeur odontogène bénigne à caractère agressif qui nécessite un diagnostic précoce et un traitement adéquat. Son site habituel est la mandibule et la chirurgie radicale demeure le traitement de référence. Nous rapportons le cas d’un améloblastome à localisation sinusienne maxillaire localement très avancé que nous avons traité par radiothérapie conformationnelle avec modulation d’intensité. L’évolution a été marquée par une rémission de la maladie avec un recul de 24 mois. La localisation maxillaire d’un améloblastome est rarement décrite, diagnostiquée à un stade tardif le plus souvent où la chirurgie optimale est impossible. A travers cette observation nous démontrons que la radiothérapie constitue une véritable alternative thérapeutique dans les formes évoluées et inopérables des améloblastomes.

## Abstract (en)

Ameloblastoma is a benign aggressive odontogenic tumor which requires early diagnosis and appropriate treatment. It commonly affects the mandible and radical surgery is the gold standard treatment. We report the case of a patient with ameloblastoma in extremely advanced phase affecting the maxillary sinus who was treated with intensity modulated conformal radiation therapy. Patient’s evolution was marked by complete remission maintained after 24 months follow-up. Maxillary ameloblastoma is not well documented in the literature. It is usually diagnosed at the later stage when optimal surgery cannot be performed. This case study aimed to demonstrate that radiation therapy is a real therapeutic alternative in the treatment of advanced and inoperable forms of ameloblastoma.

## Introduction

L’améloblastome est une tumeur bénigne odontogène rare à caractère agressif qui se localise le plus souvent au niveau mandibulaire. Les localisations maxillaires supérieures sont rarement décrites. Son évolution est lente mais se caractérise par la fréquence des récidives essentiellement après une chirurgie conservatrice [1]. Nous rapportons un cas particulier d’un patient présentant un améloblastome du sinus maxillaire traité par radiothérapie avec modulation d’intensité. A travers cette observation nous illustrons l’intérêt de la radiothérapie dans le traitement des améloblastomes.

## Patient et observation

**Figure 1:**
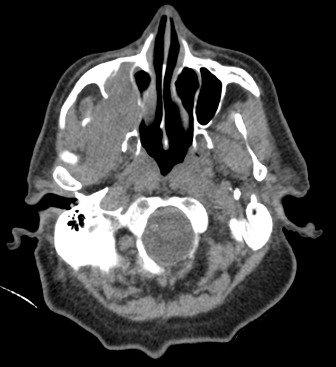
Volumineux processus du sinus maxillaire droit

**Figure 2:**
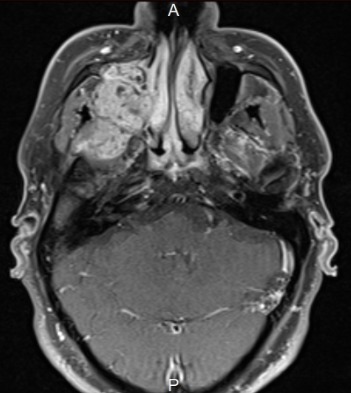
IRM de la masse tumorale

**Figure 3:**
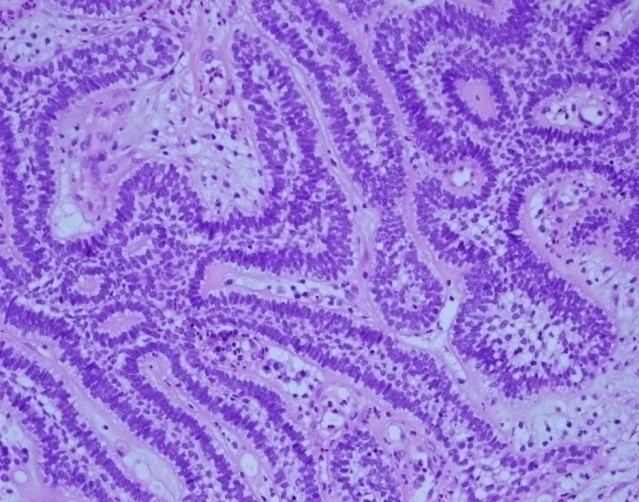
Etude anatomopathologique objectivant un améloblastome sans signe de malignité

**Figure 4:**
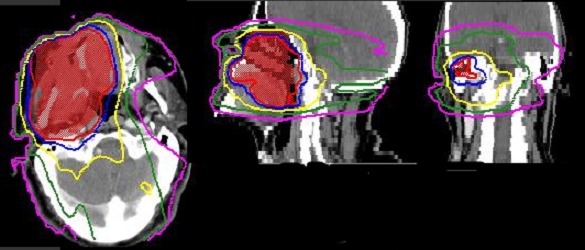
Distribution de la dose par IMRT

Il s’agit d’un patient de 73 ans, qui a consulté pour une lésion intra-buccale saignante évoluant depuis deux ans. Le scanner cervico-faciale avait montré un volumineux processus du sinus maxillaire droit avec infiltration de la muqueuse jugale et l’arcade alvéolaire droite. Ce processus tumoral mesure 65mm de grand axe avec une extension aux muscles masticateurs et à l’oropharynx Figure 1. Le bilan a été complété par une IRM qui a confirmé l’importance de l’extension tumorale Figure 2. La biopsie et l’étude anatomopathologique a objectivé un améloblastome sans signe de malignité Figure 3. Ce cas a été discuté en réunion de concertation pluridisciplinaire, l’exérèse radicale était techniquement difficile vu la taille tumorale et la proximité des organes critiques d’où un traitement à base de radiothérapie a été retenue. L’irradiation a été faite par une radiothérapie conformationnelle avec modulation d’intensité à la dose de 60Gy, 2Gy/séance et 5 séance /semaine au photon X de haute énergie 6MV. Ceci a permet une meilleur conformation de la dose à la masse tumorale avec une bonne préservation des organes à risque Figure 4.

L’évolution a été marquée par une bonne réponse clinique et radiologique. De même aucune complication n’a été rapportée. Une IRM de contrôle faite à trois mois de la fin de la radiothérapie a conclu à une diminution très remarquée de la masse tumorale. Le suivi a consisté en un examen clinique trimestriel et la réalisation régulière d’IRM de contrôle semestrielle qui ont confirmé l’absence de récidive locale clinique et radiologique avec un recul de 24 mois après la fin de la radiothérapie.

## Discussion

L’améloblastome est une tumeur odontogène bénigne à caractère agressif et potentiellement récidivante. Représente 1% des tumeurs maxillaires [2]. L’âge médian de survenue est de 35 ans, avec une légère prédominance féminine. Le maxillaire inferieur est atteint dans 80% des cas [3]. Les circonstances de découverte sont dominées par les déformations faciales et les chutes dentaires. La tumeur est indolore dans la majorité des cas. L’image radiologique la plus caractéristique est celle des « bulles de savon », traduisant une destruction osseuse poly géodique soufflant la corticale osseuse [4].

Le risque de récidive est de l’ordre de 50 à 72 % des cas. Il est majeur après une chirurgie conservatrice. Le traitement radical consiste en une exérèse large avec des marges saines de 1,5 à 2 cm. Entraînant des taux de contrôle local dépassant 90% [5]. Sauf que cette chirurgie optimale n’est pas toujours possible surtout que le diagnostic se fait à des stades tardifs notamment dans les formes maxillaires.

En revanche, la radiothérapie a trouvé aussi sa place dans le traitement des améloblastomes. Reynolds a publie le premier cas d’améloblastome irradié et il a proposé la radiothérapie pour les tumeurs localement avancées non opérables ou en cas de refus de la chirurgie [6]. Ainsi qu’en 1984 Atkinson a publié une série de patients traités par radiothérapie avec une bonne évolution [7]. Depuis lors, d’autres observations sur le rôle de la radiothérapie dans le traitement curatif de l’améloblastome ont été publiés concernant des cas d’améloblastome mandibulaire ayant bien répondu à une radiothérapie externe de 60Gy [8].

Nous présentons à travers ce travail un cas d´améloblastome du sinus maxillaire localement avancés que nous avons traité par une radiothérapie externe avec une dose de 60Gy à raison de 2Gy/séance et 5 séance /semaine au photon X de haute énergie 6MV. Et pour une meilleur optimisation de traitement nous avons utilisé la technique RCMI (radiothérapie avec modulation de l’intensité) ce qui a permet une bonne réponse tumorale avec une meilleur tolérance de la radiothérapie par les organes à risque malgré la taille tumorale et sa localisation. Cette observation, démontre l’intérêt de la radiothérapie dans les formes non opérables de l’améloblastome surtout avec l’utilisation d’une technique adaptée telle que la radiothérapie conformationnelle par modulation d’intensité.

## Conclusion

Bien que les cas d’améloblastome traités par radiothérapie soit rare la radiothérapie retrouve sa place dans les tumeurs localement avancées ou en cas de refus de la chirurgie. La Radiothérapie conformationnelle par modulation d’intensité est une réelle avancée thérapeutique qui permet de délivrer la dose adéquate à la tumeur tout en protégeant les organes à risque de voisinage.
